# Can Views and Contact with Nature at Home Help Combat Anxiety and Depression during the Pandemic? Results of the GreenCOVID study

**DOI:** 10.1002/brb3.2875

**Published:** 2023-01-30

**Authors:** Marco Garrido‐Cumbrera, Alicia González‐Marín, José Correa‐Fernández, Olta Braçe, Ronan Foley

**Affiliations:** ^1^ Health & Territory Research (HTR) Universidad de Sevilla Seville Spain; ^2^ Department of Geography Maynooth University Maynooth Ireland

**Keywords:** anxiety, depression, COVID‐19 pandemic, lockdown, Spain

## Abstract

**Background:**

The COVID‐19 pandemic and the lockdown measures have had important consequences on the mental health of the population, although little is known about the role played by nature and its benefits.

**Objectives:**

The present study aims to evaluate the risk of anxiety and depression during the first wave of the COVID‐19 pandemic in Spain and to identify the factors most strongly associated with anxiety and depression, including sociodemographic, household characteristics, and access to or contact with natural environment.

**Methods:**

GreenCOVID is an online cross‐sectional study promoted by the Health & Territory Research (HTR) of the University of Seville in Spain, Maynooth University in Ireland, and the University of Winchester in the United Kingdom. This study includes only data from Spain which were collected between April 8, 2020 and April 27, 2020. Binary logistic regression was conducted to identify the factors associated with anxiety and depression through the HADS scale.

**Results:**

Of the total of 2,464 adults who participated in GreenCOVID Spain, mean age was 38.1 years, 72.6% were female, 58.1% were at risk of anxiety, and 32.3% of depression. In the multivariable logistic regression, the factors associated with risk of anxiety were female: gender, being a student and problems at home. Regarding the risk of depression, the factors most associated were being a student, female gender, problems at home, worse evaluation of views from home and less help from outside views to cope with lockdown.

**Conclusions:**

Our findings show that during COVID‐19 pandemic, in addition to sociodemographic factors female gender and being a student, problems at home, lack of natural elements in the home, and worse appreciation of views from home were associated with mental health problems. Thus, housing conditions and access to the natural environment were important for mental health during COVID‐19 lockdown.

## Graphical abstract

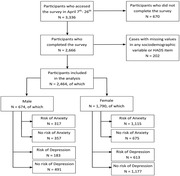

## 1 INTRODUCTION

The severe acute respiratory syndrome coronavirus 2 (SARS‐Cov‐2), a new type of coronavirus, spreads rapidly from person to person with lethal effects (Wang et al., 2020). On March 11, 2020, the World Health Organization declared the novel coronavirus (COVID‐19) outbreak a global pandemic (World Health Organization, 2020), while the virus began to spread from Wuhan (China) to the European continent hitting first Italy and, subsequently, Spain causing the highest mortality rates in Europe (Ceylan, 2020). As a way to slow down the spread of the virus, several governments, health specialists, media, and other social workers encouraged confinement and non‐attendance of gatherings (McCloskey et al., 2020). During these lockdowns in the first wave, different restriction measures were imposed, varying from country to country, affecting social distancing and mobility behaviors (Badr et al., 2020; Roy et al., 2020). These containment measures were intended to save time so that hospitals could respond to the increase in cases (Lau et al., 2020), but required people to remain in their homes and to maintain social contact with friends, family and colleagues remotely (European Centre for Disease Prevention & Control, 2020).

Confinement and deprivation of social contact produce negative psychological effects, including stress, confusion, and anger (Dubey et al., 2020; Kimhi et al., 2020; Ozamiz‐Etxebarria et al., 2020). Several studies conducted around the world have revealed a worsening of mental health of the adult general population during the COVID‐19 pandemic. In China, the first country affected by the SARS‐CoV‐2 pandemic, Wang et al. (2020) found how 28.8% of the population presented anxiety symptoms and 16.5% depressive symptoms, and women and students were associated with higher levels of anxiety and depression, using the DASS‐21 scale. Another study by Huang & Zhao (2020) found that 35.1% of the Chinese population had a prevalence of anxiety and 20.1% had depressive symptoms. In a study among 7,143 university students in China, 24.9% experienced anxiety during the pandemic (Cao et al., 2020). In India, Roy et al. (2020) noted anxiety and concern levels were high with perceived need for mental health care seen in over 80% of participants, using non‐validated Likert scales. In Israel, Kimhi et al. (2020) found that Israeli Arabs reported a higher level of distress and a lower level of resilience and well‐being compared to Israeli Jews, using the Brief Symptom Inventory. In Canada, IPSOS MORI (2020) reported that 11% respondents were conserned about anxiety and 7% about depression. Fancourt et al. (2020) found that 24.4% of the British population had moderate‐severe anxiety, and 31.4% moderate‐severe depression. In a Kaiser Family Foundation (KFF) survey carried out in the United States (April 2020), 57% of women with children at home had negative mental health problems due to the pandemic situation compared to 32% of men (Panchal et al., 2020). A study conducted in Greece showed that 73% of students increased anxiety levels and 60% increased depression levels (Kaparounaki et al., 2020).

Contact with green spaces has been associated with improved mental health (Alcock et al., 2014; Astell‐Burt et al., 2014; Douglas et al., 2017; Hartig et al., 2014) as a protective factor for anxiety and depression (Gascon et al., 2018). Direct contact with natural environments can be determined by visits to green spaces (White et al., 2013), residential proximity (Gascon et al., 2015) or neighborhoods with more green spaces. Furthermore, the use of one's yard/garden and happiness was marginally positively associated over time (Korpela et al., 2017). However, indirect exposure to nature does not require physical presence and includes views of nature through a window (Keniger et al., 2013). Several studies have shown that views of green and blue spaces improve people's mental health and well‐being (Braçe et al., 2020; Garrett et al., 2019; Kaplan, 2001; Nutsford et al., 2016), promote general health (Honold et al., 2016; Maller et al., 2006), and reduce the level of stress (Ulrich, 1979). In addition, for children, specifically girls, green spaces located immediately outside the home can help them to lead a more effective and self‐disciplined life (Taylor et al., 2002). In this sense, contact with nature, both direct and indirect, helped people to cope with the impact caused by the COVID‐19 pandemic, especially those who were under strict lockdown (Pouso et al., 2020). In a study conducted in Bulgaria with 323 students, it was detected that having vegetation visible from home or in the neighborhood environment was associated with reduced symptoms of depression and anxiety (Dzhambov et al., 2020). Results supporting this hypothesis were also obtained in Spain, where people with accessible outdoor spaces and views of blue‐green elements had positive feelings or emotions compared to those who did not have these stimuli (Pouso et al., 2020). Thus, it is proven that the natural environment has implications both in the development of common diseases and in psychological disorders associated with the pandemic situation. In contrast, the study by Pérez‐Urrestarazu et al. (2020) suggested that having plants outdoors was associated with improved well‐being during the confinement period. In addition to having implications for anxiety and depression, the frequency of green space use and the existence of views of green space from the windows of the home were associated with higher levels of self‐esteem and a reduced sense of loneliness during the pandemic (Soga et al., 2020).

Although these studies provide evidence of the impact of the pandemic on the mental health of the population, their results are not yet conclusive, and further research is needed to measure the prevalence of mental disorders and to identify the most important characteristics affecting mental health. In addition, these studies incorporate few, if any factors related to household characteristics and outdoor contact during home confinement into the assessment of mental health disorders. In the case of the Spanish population analyzed in this study, it is worth mentioning that initially the novel coronavirus SARS‐CoV‐2 attracted little attention in Spain. However, after the pandemic declaration, Spain reached one of the highest burdens of COVID‐19 across Europe, with a high prevalence of infections and deaths (Soriano et al., 2021). According to the European Commission, Spain had, in March 2020 the second largest number of positive cases in the EU‐27 (European Comission, 2020). To mitigate the pandemic curve of COVID‐19 pandemic, the Spanish government declared a 15‐day national emergency, starting on March 15; these were probably the strictest measures on the entire European continent at the time (Legido‐Quigley et al., 2020; Saez et al., 2020). Most of the population was required to remain totally confined, prevented from going outside from March 15, 2020 to April 27, 2020, affecting more than 46.9 million people (de la Cámara et al., 2020). As a result of the imposed lockdown, the spread of COVID‐19 was contained and the incidence, hospital admission, and mortality rates were reduced (Siqueira et al., 2020), so these measures were in the right direction (Mitjà et al., 2020). The aim of the present study was to evaluate the risk of anxiety and depression during the COVID‐19 lockdown in a large sample in Spain and to identify the factors most strongly associated with anxiety and depression, including sociodemographic, household characteristics, and outdoor contact. Therefore, the following hypotheses will be investigated:

1. The risk of anxiety and depression was prevalent among the general population in Spain, during the COVID‐19 pandemic.
2. There are differences in sociodemographic and household characteristics according to the risk of anxiety and depression among the general population in Spain, during the COVID‐19 pandemic.
3. Sociodemographic and household characteristics may influence the risk of anxiety and depression among the general population in Spain, during the COVID‐19 pandemic.
4. Nature contact and appreciation of nature may affect the risk of anxiety and depression among the general population in Spain, during the COVID‐19 pandemic.

## 2 MATERIALS AND METHODS

### 2.1 Survey

**FIGURE 1:**
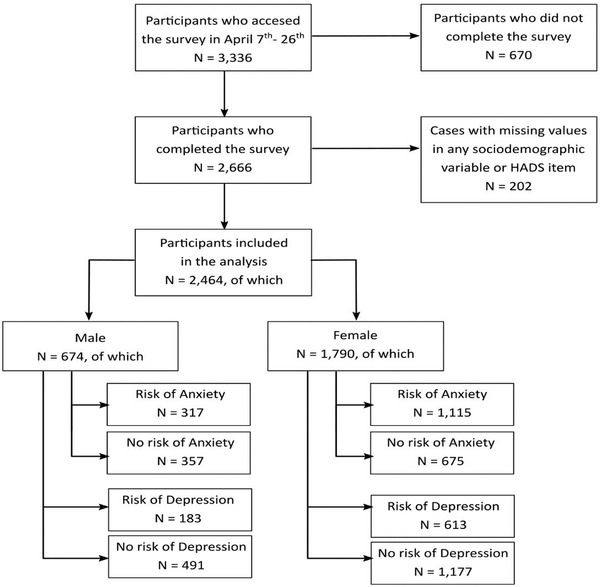
Study sample selection flowchart and sample distribution by gender and risk of anxiety and depression

GreenCOVID was an observational cross‐sectional study based on an online survey with the aim of assessing the impact of the COVID‐19 lockdown on the psychological well‐being and mental health in the general population in three European countries: Spain, Ireland, and the United Kingdom, although only the results of the Spanish survey were analyzed in this study. The GreenCOVID study was conducted by the Health & Territory Research (HTR) group of the University of Seville. The members of HTR designed the questionnaire including the following areas: sociodemographic, household, life habits, outdoor contact, well‐being, health, and mental health. Recruitment was carried out by the University of Seville press office and the Spanish Association of Geographers (AGE), gathering information from 2,666 unselected adults over the age of 16 years from the general population of Spain who participated in the survey from April 8, 2020 to April 27, 2020. Survey participants who did not respond to at least 70% of the questionnaire were excluded from the analyzed sample in order to evaluate a more robust sample with a reduced percentage of missing values. After data cleaning, 2,464 respondents who met the condition of fully responding to the sociodemographic variables and to the HAD scale were included in the analyses (Figure 1).

### 2.2 Variables

Table 1 presents a description of the variables used in the analyses, classified into three groups:

**TABLE 1:** Description of the variables used in the study

**(1) Sociodemographic characteristics**	**‐ Age** (in years) **‐ Gender** (Male/Female) **‐ Educational level** (Uneducated/Primary schooling/Secondary schooling/High school/University) **‐ Job status** (Employed/Unemployed/Student/Retired‐Early Retirement/Homemaker/Incapacity for work/Absence Leave from work/Labor force adjustment plan)
**(2) Mental health**	**‐ Anxiety** (At risk/No risk)
	**‐ Depression** (At risk/No risk)
**(3) Household characteristics and outdoor contact**	**‐ Type of residence** (Flat or apartment/Semidetached house or detached/Individual chalet/House)
	**‐ Use of outdoor spaces or window views** (Yes/No)
	**‐ Type of view from home** (No view/Inner courtyard, streets or open fields/Green or blue spaces)
	**‐ Views of natural elements from the home** (Yes/No)
	**‐ Evaluation of views from home** (0–10)
	**‐ Outdoor views help in coping with lockdown** (0–10)
	**‐ Presence of elements of nature in the home** (Yes/No)
	**‐ Problem at home** (Yes/No)

### 2.3 Mental health measures

The Hospital Anxiety and Depression Scale (HADS) is a self‐administered 14‐item scale including two subscales divided into 7 items each, on a Likert scale 0–3. HAD anxiety scores are based on odd items, HAD depression scores on even items, with a range of scores in each subscale of 0–21. Higher scores indicate a greater level of anxiety and depression. For both subscales, scores above eleven would indicate “case”, above eight would be considered “probable case”, and below eight “no case” (Zigmond & Snaith, 1983). The HADS scale has been validated in the Spanish population with good psychometric properties for the detection of psychiatric disorders (Herrero et al., 2003). For the present study, the participants were divided into two groups: people at risk of anxiety and/or depression (case and probable case) and people at no risk of anxiety and/or depression (no case).

### 2.4 Statistical analysis

The participants were divided into people at risk of anxiety and/or depression (case and probable case) and at not risk (no case). For hypotheses 1, descriptive analysis was used presenting the percentage for qualitative variables and the mean (±SD) for quantitative variables. For hypotheses 2, the Mann‐Whitney test and Pearson's chi‐square test were used to assess possible relationships with the risk of anxiety and depression (for quantitative and qualitative variables respectively). For hypotheses 3 and 4, binary logistic regressions were performed for each of the independent variables (age in years, female gender, students, not use of outdoor spaces or window views, green or blue view from home, no view of natural elements from the home, evaluation of views from home (0–10), outdoor views help in coping with lockdown (0–10), not presence of elements of nature in the home and any problem at home) against the dependent variables risk of anxiety and risk of depression. Finally, all independent variables were entered into the multiple logistic regression model. Odds Ratios (ORs) and 95% Confidence Intervals (CI) were shown in the binary logistic regression, at a significance level of 0.05.

All statistical analyses were performed using SPSS version 26.0, significance was set at *p* < .05.

## 3 RESULTS

**Results for hypotheses 1**: the mean age of our study population was 38.1 years, 72.6% were female, 70.7% had university education, and 48.5% were employed. 98.2% responded to the survey during the complete lockdown. According to the HADS (Cronbach's alpha = .719), 58.1% were at risk of anxiety (27.2% borderline cases and 30.9% cases) and 32.3% at risk of depression (7.5% borderline cases and 24.8% cases). 72.1% of participants resided in a flat or apartment, 82.0% used outdoor spaces or had windows with views of the outdoors, and 33.9% had views of green or blue spaces. The majority had views of natural environments from the home (84.9%), with an average view rating of 5.6 out of 10, helping them to cope with the lockdown. Within the home, 67.8% had natural elements and 40.6% had some sort of identifiable problems in the home (Supplementary Table S1).

**Results for hypotheses 2**: risk of anxiety was presented among younger (36.8 vs. 39.8 at not risk; *p* < .001), and females (62.3% vs. 47.0% of males; *p* < .001). In addition, 67.7% of students and 64.3% of homemakers were at risk of anxiety (vs. 53.7% of employees). For depression, those at risk were younger (35.6 vs. 39.2; at not risk *p* < .001), and females (34.2% vs. 27.2% of males; *p* = .001). Moreover, 42.2% of students and 38.1% of homemakers were at risk of depression (vs. 27.5% of employees). Further information on the associations between sociodemographic characteristics and the risk of anxiety and depression can be found in the Supplementary Table S2).

Overall, a higher proportion of people at risk of anxiety had worse views from home (p < 0.001), had no views of nature elements from home (p = 0.029), reported worse evaluation of views from home (p < 0.001), that the views did not help them cope with lockdown (p < 0.001), and reported problems at home (p < 0.001). On the other hand, a higher proportion of people at risk of depression did not use outdoor spaces and views from windows (p < 0.001), did not have views of green or blue spaces (p < 0.001), did not have views of nature elements from home (p < 0.001), reported worse evaluation of views from home (p < 0. 001), that views did not help them cope with lockdown (p < 0.001), had no natural elements at home (p = 0.006), and reported problems at home (p < 0.001; Supplementary Table S3).

**Results for hypotheses 3 and 4**: from univariable logistic regression, the risk of anxiety was associated with younger age (OR = 0.982), female gender (OR = 1.860), students (OR = 1.670), lack of views of green and blue spaces (OR = 0.709), lack of views of elements of nature (OR = 1.289), poorer evaluation of views from home (OR = 0.925), less help from outdoor views in coping with lockdown (OR = 0.950), and problems at home (OR = 1.540). When all variables were entered into the multivariable logistic regression model, the factors most associated with risk of anxiety were female gender (OR = 1.780), students (OR = 1.429), and the presence of problems at home (OR = 1.343) (Table 2).

**TABLE 2:** Binary logistic regression to predict the factors associated with the risk of anxiety during the COVID‐19 lockdown (*N* = 2,445)

	Univariable logistic regression			Multivariable logistic regression		
Variable	OR^†^	CI (95%)	p‐value	OR^†^	CI (95%)	p‐value
**Age**	0.982	0.976, 0.988	**<.001**	0.996	0.988, 1.004	.293
**Gender** (Female)	1.860	1.556, 2.225	**<.001**	1.780	1.479, 2.142	**<.001**
**Job status** (Student)	1.670	1.356, 2.058	**<.001**	1.429	1.100, 1.856	**.008**
**Type of view from home** (Green or blue spaces)	0.709	0.600, 0.839	**<.001**	0.853	0.699, 1.042	.120
**Views of natural elements from the home** (No)	1.289	1.027, 1.619	**.029**	1.032	0.800, 1.330	.810
Evaluation of views from home (0–10)	0.925	0.898, 0.953	**<.001**	0.976	0.934, 1.019	.267
Outdoor views help in coping with lockdown (0–10)	0.950	0.927, 0.973	**<.001**	0.978	0.950, 1.008	.153
**Problems at home** (Yes)	1.540	1.305, 1.819	**<.001**	1.343	1.126, 1.601	**.001**

^†^ OR < 1 indicates a decrease in the likelihood of risk of anxiety; OR > 1 indicates an increase in the likelihood of risk of anxiety. P‐values are shown in bold due to statistical significance (p<0.05).

OR = odds ratio; CI = confident interval.

From univariable logistic regression, the risk of depression was associated with younger age (OR = 0.978), female gender (OR = 1.397), students (OR = 1.717), not use of outdoor spaces or windows (OR = 1.858), lack of views of green and blue spaces (OR = 0.583), lack of views of elements of nature (OR = 1.608), poorer evaluation of views from home (OR = 0.848), less help from outdoor views in coping with lockdown (OR = 0.894), not presence of natural elements in the home (OR = 1.283), and problems at home (OR = 1.797). When all variables were entered into the multivariable logistic regression model, the qualitative factors most associated with risk of depression were being students (OR = 1.330), female gender (OR = 1.308), and the presence of problems at home (OR = 1.403), while the quantitative factors most associated with the risk of depression were worse evaluation of views from home (OR = 0.917), and less help from views to the outside to cope with lockdown (OR = 0.949) (Table 3).

**TABLE 3:** Binary logistic regression to predict the factors associated with the risk of depression during the COVID‐19 lockdown (*N* = 2,444)

	Univariable logistic regression			Multivariable logistic regression		
Variable	OR^†^	CI (95%)	p‐value	OR^†^	CI (95%)	p‐value
**Age**	0.978	0.971, 0.985	**<.001**	0.992	0.983, 1.001	.082
**Gender** (Female)	1.397	1.149, 1.700	**.001**	1.308	1.065, 1.606	**.011**
**Job status** (Student)	1.717	1.401, 2.103	**<.001**	1.330	1.020, 1.734	**.035**
**Use of outdoor spaces or window views** (No)	1.858	1.505, 2.293	**<.001**	1.181	0.930, 1.500	.171
**Type of view from home** (Green or blue spaces)	0.583	0.484, 0.701	**<.001**	0.926	0.742, 1.155	.493
**Views of natural elements from the home** (No)	1.608	1.283, 2.016	**<.001**	1.028	0.795, 1.329	.835
Evaluation of views from home (0–10)	0.848	0.821, 0.876	**<.001**	0.917	0.876, 0.960	**<.001**
Outdoor views help in coping with lockdown (0–10)	0.894	0.871, 0.916	**<.001**	0.949	0.920, 0.98	**.001**
**Presence of elements of nature in the home** (No)	1.283	1.073, 1.534	**.006**	1.036	0.851, 1.260	.727
**Problems at home** (Yes)	1.797	1.514, 2.134	**<.001**	1.403	1.167, 1.685	**<.001**

^†^ OR < 1 indicates a decrease in the likelihood of risk of depression; OR > 1 indicates an increase in the likelihood of risk of depression. P‐values are shown in bold due to statistical significance (p<0.05).

OR = odds ratio; CI = confident interval.

## 4 DISCUSSION

Based on the analyses performed, our study confirmed the following hypotheses:

1. The risk of anxiety and depression was prevalent among the general population in Spain, during the COVID‐19 pandemic.
2. There are differences in sociodemographic and household characteristics according to the risk of anxiety and depression among the general population in Spain, during the COVID‐19 pandemic.
3. Sociodemographic and household characteristics may influence an increased risk of anxiety and depression among the general population in Spain, during the COVID‐19 pandemic.
4. Nature contact and appreciation of nature contributed to decreased risk of anxiety and depression among the general population in Spain, during the COVID‐19 pandemic.

Our study shows that, during the complete lockdown, a high proportion of the Spanish population were at risk of anxiety (58.1%) or at risk of depression (32.3%). The factors most associated, in the multivariable regression analysis, with anxiety and depression were being female, student, or experiencing the presence of problems at home during COVID‐19. In the univariable logistic regression, not having views of green or blue spaces, a lack of elements with outdoor contact, and a poorer appreciation of views from the home were individually associated with the risk of anxiety and depression. Specifically, not using outdoor spaces or windows and less help from views to the outside to cope with lockdown were associated with the risk of depression. However, this association was not found for anxiety risk.

In our study, we were able to identify several factors associated with an increased risk of anxiety and depression during lockdown, highlighting gender differences. These results are in line with other studies such as Ausín et al. (2020) where it is found that women suffered a greater impact on mental health, loneliness, and well‐being than men. These high levels of anxiety and depression risk are consistent with other studies such as Fancourt et al. (2020) in which high percentages of anxiety (24.4%) and depression (31.4%) were observed during COVID‐19 lockdown or another by Roy et al. (2020) in which anxiety levels were high. A longitudinal study conducted in Spain during COVID‐19 lockdown, using the Depression Anxiety and Stress Scale (DASS‐21) and the Impact of Event Scale (IES), confirmed an increase in anxiety, depression and stress scores, specially among younger (Planchuelo‐Gómez et al., 2020).

Females and students were at higher risk of anxiety and depression during the lockdown, as in the study by González‐Sanguino et al. (2020) where Spanish women and students experienced higher levels of anxiety, depression, and post traumatic stress disorder (PTSD) during the lockdown. This may be due to women playing different roles at home and students being forced to follow classes virtually. Other studies such as the literature review conducted by Sepúlveda‐Loyola et al. (2020) that incorporated 41 studies where they evaluated how quarantine had affected the mental health of the population and the most repeated risk factor was being a woman.

Furthermore, our results are in line with Braçe et al.’s (2020) study, in which views of green spaces were associated with a lower risk of anxiety and depression. Moreover, as Pouso et al.’s (2020) study showed, contact with nature helped people cope with the impact caused by the COVID‐19 pandemic, especially those who were under strict confinement, which is consistent with our findings.

Other studies have evaluated home characteristics such as the lack of a balcony or poor‐quality views, relating these characteristics to moderate‐severe and severe depressive symptoms (Amerio et al., 2020). As expressed by Rautio et al. (2018) in a systematic review relating depression to the living environment, lack of green spaces, home characteristics, and views were related to the risk of depression.

With respect to the use of outdoor spaces of the dwellings, the present findings seem to be consistent with other research carried out in Spain, such as Cuerdo‐Vilches et al. (2020), which assessed the use of outdoor spaces and the relationship with natural elements, showing an over 70% of Spaniards used outdoor spaces and had positive relationships with natural elements.

Considering the latest developments, researchers should incorporate the use of new technologies to be able to assess the impact of interaction with nature. This could be done by providing images of nature or green areas or even using virtual reality (Chan et al., 2021; Gao et al., 2019).

This study also has limitations. The impossibility of carrying out face‐to‐face surveys due to the confinement led to an online study. This has meant that we have fewer responses from elderly people, the most vulnerable to COVID‐19 in terms of both health and socialization (Sepúlveda‐Loyola et al., 2020). For this reason, we consider it necessary to study the effects of the COVID‐19 pandemic in elderly people in nursing homes, since the mortality rate has been four times higher than that of the general population (Siqueira et al., 2020). Another limitation is that not all independent variables were significant for anxiety and depression in the multivariable regression models. In this regard, not using outdoor spaces or windows and less help from views to the outside to cope with lockdown were only associated with the risk of depression and not with risk of anxiety. Additionally, due to the cross‐sectional nature of the study, it is not possible to establish cause‐effect relationships and, therefore, the results should be interpreted with caution. Finally, another limitation is the lack of assessment of other prevalent mental disorders, such as bipolar disorder, post traumatic stress disorder (PTSD), schizophrenia or eating disorders, which affect millions of people worldwide (World Health Organization, 2022).

Our study has not evaluated how the population is affected by the impossibility of accessing green spaces during the lockdown period but according to Ugolini et al. (2021), this sense of loss can be alleviated through a green view from the window or a view of a wider natural landscape.

There are several factors that could have influenced the pandemic to be worse in Spain than in other European countries. First, Spain has the second‐highest proportion of people living in flats/apartments in Europe (64.9%) compared to the 46% of the EU population (Eurostat, 2020), making interaction with the outside world more difficult. Secondly, Spain is a Latin country where citizens prefer closer social interaction than individuals from North America or Northern Europe (Varea et al., 2018). Moreover, the economic crisis of 2008 in Spain led to an increase in the prevalence of mental disorders, higher than in northern European countries (Salvador‐Carulla & Roca, 2013). Therefore, compared to other EU countries, the pandemic has had a greater effect in Spain, so that economic recovery and normality in social relations will also be slower.

Although several studies have evaluated the prevalence of anxiety and/or depression during COVID‐19 lockdown (Kimhi et al., 2020; Ozamiz‐Etxebarria et al., 2020), in our study, in addition to analyzing the risk of anxiety and depression, we identified what factors are most associated with the risk of anxiety and depression, including household characteristics and outdoor contact. It is important to highlight that data were collected for our study during the strictest period of COVID‐19 lockdown when most of the population was prevented from going outside their homes. Another strength of our study is its large sample size from all the 17 Autonomous Communities leading to high representativeness. In addition, the use of a validated measurement HADS scale to assess the risk of anxiety and depression ensures reliability and validity (Herrero et al., 2003; Zigmond & Snaith, 1983). Our aim, set at the beginning of the research, was finally achieved as we identified the factors more associated with the risk of anxiety and depression during the COVID‐19 lockdown. The present assessment of the prevalence of anxiety and depression and its associated factors during lockdown may provide relevant information for health planners, policymakers, and environmental psychologists.

## 5 CONCLUSION

During the beginning of the COVID‐19 pandemic, more than half of the Spanish population were at risk of anxiety and a third at risk of depression. Our findings show that the most associated factors to predict the risk of anxiety and depression were female gender, being student, and presence of problems at home. In addition, individually, the lack of natural space in the home, the lack of views from the home, and a poorer appreciation of views were associated with poor mental health during the home confinement. These results confirm that the strict measures imposed by the Spanish government, in addition to bending the curve of COVID‐19 infections, have had a significant impact on the mental health of the population, with women, students, and those with problems at home most at risk. However, the views and contact with elements of the environment could help people's mental health in the current COVID‐19 pandemic.

## FUNDING

This research did not receive any specific grant from funding agencies in the public, commercial, or not‐for‐profit sectors.

## CONFLICT OF INTEREST

The authors declared no potential conflicts of interest with respect to the research, authorship, and/or publication of this study.

### ETHICS APPROVAL STATEMENT

Ethics Committees of the University of Maynooth (Ireland) and the University of Winchester (UK). For Spain not available.

### PATIENT CONSENT STATEMENT

All participants reported informed consent.

### PEER REVIEW

The peer review history for this article is available at https://publons.com/publon/10.1002/brb3.2875.

## Supporting information

Supplementary Table S1. Sample characteristics, household characteristics, and outdoor contact during the COVID‐19 lockdown (*N* = 2,464, unless specify)
Supplementary Table S2. Sociodemographic characteristics of the risk of anxiety and depression during the COVID‐19 lockdown (*N* = 2,464)
Supplementary Table S3. Household characteristics according to the risk of anxiety and depression during the COVID‐19 lockdown (*N* = 2,464)

## Data Availability

The data that support the findings of this study are available from the corresponding author upon reasonable request.
