# Supplementary material for: Can Views and Contact with Nature at Home Help Combat Anxiety and Depression during the Pandemic? Results of the GreenCOVID study
Source: Brain Behav. 2023 Jan 30;13(3):e2875. doi: 10.1002/brb3.2875 (PMC10013950; doi:10.1002/brb3.2875)
Supplement: Supplementary file 1 — Supplementary Table S1. Sample characteristics, household characteristics, and outdoor contact during the COVID‐19 lockdown (N = 2,464, unless specify) Supplementary Table S2. Sociodemographic characteristics of the risk of anxiety and depression during the COVID‐19 lockdown (N = 2,464) Supplementary Table S3. Household characteristics according to the risk of anxiety and depression during the COVID‐19 lockdown (N = 2,464) [file BRB3-13-e2875-s001.docx]

**Supplementary tables**

|  | **Mean ± SD or n (%)** |
| --- | --- |
| **Age** (Years) | 38.1 ± 12.9 |
| **Gender** (Female) | 1,790 (72.6) |
| **Educational level** (University) | 1,742 (70.7) |
| **Job status** (Employed) | 1,194 (48.5) |
| **Risk of anxiety** (HADS) | 1,432 (58.1) |
| **Risk of depression** (HADS) | 796 (32.3) |
| **Type of residence** (Flat/apartment) | 1,777 (72.1) |
| **Use of outdoor spaces or window views** (Yes) | 2,021 (82.0) |
| **Type of view from home** (Green or blue spaces) | 835 (33.9) |
| **Views of natural elements from the home** (Yes) | 2,091 (84.9) |
| **Evaluation of views from home (0-10)** | 5.6 ± 2.7 |
| **Outdoor views help in coping with lockdown (0-10)** | 5.7 ± 3.4 |
| **Presence of elements of nature in the home** (Yes) N: 2,463 | 1,669 (67.8) |
| **Problem at home** (Yes) N: 2,445 | 993 (40.6) |

**Supplementary table 1.** Sample characteristics, household characteristics, and outdoor contact during the COVID-19 lockdown (N=2,464, unless specify)

| **Sociodemographic variables** | **Anxiety** | | **P-value** | **Depression** | | **P-value** |
| --- | --- | --- | --- | --- | --- | --- |
|  | **Risk** | **No risk** |  | **Risk** | **No risk** |  |
| **Age** | | | | | | |
| Years | 36.8 ± 12.6 | 39.8 ± 13.1 | **<0.001** | 35.6 ± 12.4 | 39.2 ± 13.0 | **<0.001** |
| **Gender** |  |  |  |  |  |  |
| Male | 317 (47.0) | 357 (53  0) | **<0.001** | 183 (27.2) | 491 (72.8) | **0.001** |
| Female | 1,115 (62.3) | 675 (37.7) |  | 613 (34.2) | 1,177 (65.8) |  |
| **Educational level** | | | | | | |
| Uneducated or primary schooling | 33 (64.7) | 18 (35.3) | 0.414 | 19 (37.3) | 32 (62.7) | 0.690 |
| Secondary schooling | 61 (64.9) | 33 (35.1) |  | 30 (31.9) | 64 (68.1) |  |
| High school | 332 (57.5) | 245 (42.5) |  | 195 (33.8) | 382 (66.2) |  |
| University | 1,006 (57.7) | 736 (42.3) |  | 552 (31.7) | 1,190 (68.3) |  |
| **Job status** | | | | | | |
| Employed | 641 (53.7) | 553 (46.3) | **<0.001** | 328 (27.5) | 866 (72.5) | **<0.001** |
| Unemployed | 171 (60.2) | 113 (41.2) |  | 99 (34.9) | 185 (65.1) |  |
| Student | 334 (67.7) | 159 (32.3) |  | 208 (42.2) | 285 (57.8) |  |
| Retired/ early retirement | 38 (45.8) | 45 (54.2) |  | 21 (25.3) | 62 (74.7) |  |
| Homemaker | 27 (64.3) | 15 (35.7) |  | 16 (38.1) | 26 (61.9) |  |
| Incapacity for work / Leave of absence from work | 60 (63.8) | 34 (36.2) |  | 33 (35.1) | 61 (64.9) |  |
| Labor force adjustment plan | 161 (58.8) | 113 (41.2) |  | 91 (33.2) | 183 (66.8) |  |

P-values are shown in bold due to statistical significance (p<0.05).

**Supplementary table 2.** Sociodemographic characteristics of the risk of anxiety and depression during the COVID-19 lockdown (N=2,464)

| **Household characteristics** | **Anxiety** | | **P-value** | **Depression** | | **P-value** |
| --- | --- | --- | --- | --- | --- | --- |
|  | **Risk** | **No risk** |  | **Risk** | **No risk** |  |
| **Type of residence** | | | | | | |
| Flat/apartment | 1,017 (57.2) | 760 (42.8) | 0.545 | 578 (32.5) | 1,199 (67.5) | 0.386 |
| Townhouse/detached | 159 (59.8) | 107 (40.2) |  | 88 (33.1) | 178 (66.9) |  |
| Individual chalet | 71 (61.2) | 45 (38.8) |  | 29 (25.0) | 87 (75.0) |  |
| House | 185 (60.7) | 120 (39.3) |  | 101 (33.1) | 204 (66.9) |  |
| **Use of outdoor spaces or window views** | | | | | | |
| Yes | 1,164 (57.6) | 857 (42.4) | 0.262 | 601 (29.7) | 1,420 (70.3) | **<0.001** |
| No | 268 (60.5) | 175 (39.5) |  | 195 (44.0) | 248 (56.0) |  |
| **Type of view from home** | | | | | | |
| No view | 4 (100.0) | 0 (0.0) | **<0.001** | 2 (50.0) | 2 (50.0) | **<0.001** |
| Inner courtyard, streets, or open fields | 988 (60.8) | 637 (39.2) |  | 587 (36.1) | 1,038 (63.9) |  |
| Green or blue spaces | 440 (52.7) | 395 (47.3) |  | 207 (24.8) | 628 (75.2) |  |
| **Views of natural elements from the home** | | | | | | |
| Yes | 1,196 (57.2) | 895 (42.8) | **0.029** | 641 (30.7) | 1,450 (69.3) | **<0.001** |
| No | 236 (63.3) | 137 (36.7) |  | 155 (41.6) | 218 (58.4) |  |
| **Evaluation of views from home (0-10)** | 5.3 ± 2.8 | 5.9 ± 2.8 | **<0.001** | 4.7 ± 2.8 | 5.9 ± 2.6 | **<0.001** |
| **Outdoor views help in coping with lockdown (0-10)** | 5.5 ± 3.5 | 6.0 ± 3.3 | **<0.001** | 4.8 ± 3.5 | 6.1 ± 3.3 | **<0.001** |
| **Presence of elements of nature in the home^1^** | | | | | | |
| Yes | 956 (57.3) | 713 (42.7) | 0.232 | 509 (30.5) | 1,160 (69.5) | **0.006** |
| No | 475 (59.8) | 319 (40.2) |  | 386 (36.0) | 508 (64.0) |  |
| **Problems at home** | | | | | | |
| Yes | 640 (64.5) | 353 (35.5) | **<0.001** | 399 (40.2) | 594 (59.8) | **<0.001** |
| No | 785 (54.1) | 667 (45.9) |  | 395 (27.2) | 1,057 (72.8) |  |

^1^Including: trees, lawns, plants, orchard, and garden; P-values are shown in bold due to statistical significance (p<0.05).

**Supplementary table 3.** Household characteristics according to the risk of anxiety and depression during the COVID-19 lockdown (N=2,464)
